# Perspectives of stakeholders on post-trial access arrangements in Ethiopia: a qualitative study

**DOI:** 10.1080/11287462.2025.2497599

**Published:** 2025-05-05

**Authors:** Gudina Terefe Tucho, Cynthia Khamala Wangamati, Diribe Makonene Kumsa, Rosemarie de la Cruz Bernabe

**Affiliations:** a Department of Environmental Health Sciences and Technology Institute of Health, Jimma University, Jimma, Ethiopia; b Centre for Medical Ethics, Institute of Health and Society, Faculty of Medicine, University of Oslo, Oslo, Norway; c Department of Sociology, College of Social Sciences and Humanities, Jimma University, Jimma, Ethiopia

**Keywords:** Post-trial access, post-trial responsibilities, benefit-sharing, clinical trials, community engagement, stakeholders

## Abstract

While there is limited practical experience and guidance on post-trial access (PTA) in clinical trials in low- and middle-income countries, the concept of benefit-sharing is firmly established in international ethical guidelines. Few studies have been conducted in sub-Saharan African on PTA despite its importance in distributive justice. This study aims to explore the stakeholders’ perspectives on PTA and its feasibility in Ethiopia. An exploratory qualitative study using in-depth interviews was conducted with 22 stakeholders involved in clinical trials study and review. Deductive thematic analysis was used to analyze the data. We found that research participants had limited knowledge on PTA. They opined that both trial participants and communities should benefit from clinical trials and multi-stakeholder collaboration was key in PTA planning and arrangements. However, they were uncertain of PTA feasibility in Ethiopia mostly due to a lack of legislation, regulations and guidelines on PTA and fear of losing sponsors because of increased costs resulting from them being obligated to provide PTA. It was recommended that Ethiopia establishes legislation and guidelines to govern PTA. Multi-stakeholder engagement in PTA planning and arrangements is key for meaningful PTA as the responsibility is shouldered by all parties.

## Introduction

The Council for International Organizations of Medical Sciences (CIOMS) guidelines described post-trial access (PTA) as the obligation of sponsors and researchers in coordination with the host government and other relevant stakeholders including the community and the research ethics committees to ensure that any intervention or product developed, and knowledge generated after a clinical trial is made available to the studied population or community promptly (CIOMS, 2016).

In the past two decades, health research in Africa has grown significantly in response to the high disease burden in the continent. This surge in clinical trials is driven by pressing health needs, weak regulatory frameworks, lower operational costs, and the availability of research participants (Mokgatla et al., 2018; Singh et al., 2019). However, this expansion could have significant ethical concerns, particularly regarding inadequate oversight, exploitation of vulnerable populations, and the failure to ensure PTA to interventions. Many African countries do not have the necessary regulatory infrastructure and independent professional competences to enforce ethical standards, leading to inconsistent trial monitoring, accountability and trial quality (Bangura et al., 2022; De Pretto-Lazarova et al., 2022).

A critical issue is the neglect of post-trial benefits for participants, with sponsors and researchers often discontinuing successful interventions after trials conclude, leaving the trial communities without access to alternative treatments they contributed to develop(Grady, 2005; Johnson, 2024). This ethical gap is exacerbated by socioeconomic disparities, weak policy enforcement, and insufficient training of research ethics committees (Bangura et al., 2022; Ng et al., 2015). For instance, a study in Ethiopia found that Research Ethics Committee (REC) members had limited capacity to review, approve, and monitor clinical trials effectively, leaving participants vulnerable to exploitation (Seralegne et al., 2024). Similar challenges have been reported across sub-Saharan Africa, where RECs struggle with inadequate resources, conflicts of interest, and a lack of legal frameworks to propose PTA (Chaudhry et al., 2022).

International ethical frameworks explicitly emphasize the importance of PTA and benefit-sharing in clinical research. The United Nations Educational, Scientific and Cultural Organization Declaration on Bioethics and Human Rights underscores the obligation to share scientific benefits, particularly with developing countries (Andorno, 2007; Ten Have & Jean, 2009). Similarly, the Declaration of Helsinki mandates PTA for all participants who may still require an intervention proven beneficial in the trial (WMA, 2013), while the CIOMS Guidelines call for multi-stakeholder collaboration to ensure interventions and knowledge are made available to host communities (CIOMS, 2016). Collectively, these guidelines affirm that PTA whether through continued drug access, equitable pricing, or dissemination of research findings must be integral to trial design.

Hence, ethical guidelines are clear that PTA, in the form of concrete plans and procedures for benefit-sharing and access (may it be in the form of continued access to the study drug after the trial completion for research participants; free or differential and equitable pricing of efficacious drug and devices approved in the host country for selected patients or for everyone; provision of knowledge generated from clinical trials, among others), must be intrinsic to clinical trial planning. Despite these ethical guidelines, the general status quo of PTA is not encouraging as the provisions on PTA in the various guidelines are characterized by disagreements and inconsistencies, resulting in the practice of PTA as “rather the exception than the rule” (Schipper & Colona, 2015). These findings align with empirical evidence from low- and middle-income countries (LMICs), where PTA remains systematically neglected. For instance, a study by Bernabe and colleagues on industry-sponsored trials in the Philippines revealed that the majority lacked formal PTA provisions plan or intention (Jimenez et al., 2019). These disparities reflect systemic challenges across LMICs, where weak regulatory enforcement and inherent power imbalances between trial sponsors and host institutions exacerbate ethical shortcomings in PTA provisions (Alemayehu et al., 2018; Paul et al., 2018). Empirical evidence further underscores that studies on maternal vaccine trials, for instance, found that PTA commitments were frequently absent or inadequately addressed in trial agreements (Van Roessel et al., 2019). This neglect is particularly alarming given the substantial rise in offshored clinical trials in LMICs over the past decade (Agency, 2013). Most clinical trial research proposal review guidelines poorly incorporated statements even regarding benefit-sharing (FMHACAE, 2017).

Despite the critical role of PTA in promoting distributive justice, empirical research on PTA practices in sub-Saharan Africa remains limited. Existing studies highlight key challenges, including the feasibility of sustaining healthcare services for trial participants in LMICs (Andanda & Wathuta, 2018), and implementation challenges of PTA plan in HIV prevention research (Paul et al., 2018). However, no prior studies have examined PTA in the Ethiopian context, where weak health systems and gaps in research governance may exacerbate ethical and logistical challenges. This study addresses this gap by exploring stakeholder perspectives on PTA and assessing its feasibility in Ethiopia, with the aim of informing ethical guidelines and trial policies in low-resource settings. Moreover, the study findings will serve as a baseline for further research involving quantitative studies and perspectives from research participants and host communities. Additionally, it aims to bring the issues of PTA to global attention and action.

## Methodology

### Study design

This study employed an exploratory qualitative study design, utilizing grounded theory methodology, to investigate PTA in Ethiopia from September 2021 to February 2022. The grounded theory approach was selected to systematically explore stakeholder perspectives, allowing for an in-depth understanding of the complex, context-specific issues surrounding PTA, previously unexplored topic in the Ethiopian setting.

### Study setting

The study was conducted in Ethiopia, with a geographic focus on Oromia regional state, the former Southern Nations, Nationalities, and Peoples’ Region, and Addis Ababa city administration. These regions were selected as they represent the major clinical trial sites in the country. Northern regions including Amhara and Tigray were excluded from the study due to ongoing security concerns and instability during the research period.

### Study participants and sampling

Twenty-two people participated in the study: 20 men and 2 women. The study participants included principal investigators conducting/ed clinical trials on Malaria, Tuberculosis, and neglected tropical diseases (NTD); funding organization (FO), institutional review boards (IRB); Regional Ethics Review Committees (RERC); National Ethics review Committee (NEC) and Regulatory Authority (RA). IRB, RERC, and NEC were considered differently in the sampling due to their specific responsibilities. In the Ethiopian context, the IRB is responsible for reviewing institutional research proposals and referring the approved proposals to the respective RERC and national REC for further review and approval. The RERC has the mandate to review and approve research proposals implemented in its region. The REC conducts the final ethical review and makes the ultimate decision. Accordingly, we interviewed eight principal investigators, six IRB members, five RERC members, one RA, one national ethics committee member, and one person from an FO. The number of study participants from the principal investigators, the IRB, and the regional ethics committee was determined based on data saturation. Purposive and snowballing methods were used to recruit the study participants from the relevant stakeholders. Snowballing methods were used mainly for recruiting researchers/principal investigators, other study participants were directly contacted at their respective working areas. In particular, we considered individuals in higher and relevant positions for participants who were directly contacted in their respective work areas. Given their extensive experience and relevant responsibilities, their responses are representative.

### Data collection

The data was collected from September 2021 to February 2022. A team of interviewers was trained by the research project team members from Jimma University on the protocol, COVID-19 preventive measures, and ethical standard operating procedures that were to be followed during the interview. Moreover, the interviewers had prior experience of qualitative data collection methods. The team of interviewers comprised three individuals (the first and third author and 2 qualitative research assistants) with specific tasks (the authors did the interviews while the research assistants took down notes and did the voice recording). Interviews were conducted using semi-structured interview guides. All interviews except for one (conducted on Zoom) were in person and audio-recorded in private spaces at the workplace of the research participants. The interviews were conducted in local languages (in Amharic and Afan Oromo and in English) and lasted between 45 and 60 minutes. The interviews were transcribed verbatim by the two research assistants. The transcripts were later translated into English by a language expert. The themes explored were awareness on PTA and its importance, clinical trial approval processes, PTA challenges, feasibility of PTA, and recommendations.

### Data analysis

Data analysis was conducted deductively; thematic analysis was used to identify, analyze, and report patterns within the data (Malterud, 1993).

The iterative thematic deductive analysis entailed familiarization with the data, generation of initial codes, themes search, themes review, and definition and naming of themes (Braun & Clarke, 2006). After data collection, transcription and translation, transcripts and field notes were read and reread several times to gain an in-depth understanding of the data and coded using ATLAS.ti version 7.0 by the first and third authors. The first and third authors then deliberated on and agreed on the developed subthemes and themes. The results were presented by including quotes from the respondents. The quotes were cited sequentially, with R1, R2, and so on representing respondents. IRB denotes respondents from the Institutional Research Ethics Board, RA refers to Regulatory Agency respondents, PI refers to participants from the principal investigator, RERC represents participants from the Regional Ethics Research Committee, NEC stands for participants from the National Ethics Committee, and FO stands for participants from FOs.

### Ethics approval

Ethical approval was obtained from the Jimma University Institute of Health Institutional Review Board and final clearance from the NEC established in the former Ministry of Science and Higher Education (MoSHE) and currently in the Ministry of Education (MoE; Ref. No: MoSHE/RD/04/246/84/21). Written informed consent was obtained from all the research participants before interview commencement. Participants were informed that their participation was voluntary and that they could withdraw anytime from the study. They were also assured of confidentiality and privacy of shared information.

## Results

The findings from the study are summarized under three main themes, namely, (1) knowledge of stakeholders on PTA arrangements, (2) experiences on clinical trial approval processes, and (3) feasibility of PTA arrangements and challenges.

### Awareness of stakeholders on PTA arrangements

We explored the stakeholders’ understanding and perception of PTA arrangements, the beneficiaries, and the reasons for the arrangements.

#### What is PTA?

Most of the study participants (75%) had a limited understanding of PTA. Many of the principal investigators shared that they did not fully understand PTA arrangements, and described PTA as engaging the community in planning and designing the clinical trial.

Some principal investigators, members of ethics review committees, regulatory authorities, and a member of an FO mainly conceptualized and described PTA as the continued use of the drug by participants after the trial’s completion. They also noted that PTA involves ensuring the drug’s accessibility following post-marketing authorization and disseminating the clinical trial findings to relevant stakeholders.

A Participant from an FO described PTA as

… People who took part in the trial continue receiving the drug after the trial has completed been and has been proven with the expected result. (R3-FO participant)

#### Who are PTA beneficiaries?

Regarding PTA beneficiaries, there were differences in opinion on who should benefit. Some participants believed that certain groups such as trial participants or people in the community affected by specific trial diseases should be PTA beneficiaries. Others, on the other hand, said that the whole community should benefit.

Participants explained:

If the trial is about a drug trial like malaria or HIV/AIDS, it must be facilitated and arranged for the patients and the population affected by the malarial and HIV/AIDS infection, thus the trial participants should benefit from the improved drugs or services as a nation or a country. (R5-PI1 participant).

… We do not think study participants should get different benefits than their community. We believe the study benefits the large community and does not harm study participants. In that case, the study participants continue taking the drug if it is new and its efficacy is proven. We think the study participants should benefit equally as the large community and do not need specially arranged benefits for them. (R1-IRB1 participant)

#### PTA plans and arrangements

In terms of plans and arrangements, participants opined that PTA arrangements are key for the benefit of the communities under study. They felt that PTA arrangements and negotiations should not only involve organizations or groups participating in the trials but the whole community with multisectoral collaboration. Participants perceived PTA as necessary based on utilizing societal goods to run clinical trials and the fact that pharmaceutical companies profit hugely from successful clinical trial products. Participants said:

PTA is not only for sector organizations or the groups participating in the trial, but it should be arranged or facilitated for all stakeholders including the communities. (R6-PI2 Participant)

In a developing country where resources are limited, drugs approved by clinical trials are not easily accessible; thus, dealing with the relevant organization on how they can be made available at a reasonable cost, and its visibility in both urban and rural areas have to be arranged and negotiated. (R7-PI3 participant)

Suppose that we conducted some studies in rural areas using the rural health facility as a public contribution. If we do not have a mechanism to provide certain access at the end of the trial, it will be clear exploitation. Even though it is not clearly demanding money, indirectly, it is taking resources from society. Therefore, we must have ways to benefit both sides (the research and the participants). (R8-IRB3 Participant)

### Experiences on clinical trial approval processes

Due to a limited understanding of PTA, we focused on the current practices of clinical trial review and approval processes to understand the feasibility of arranging for PTA.

#### Clinical trial approval processes

Approval of clinical trials involves several factors and levels of decision making. Prior to submission for the approval, the principal investigators adjust their protocols according to specific guidelines and requirements. First, the protocols are submitted to IRBs for ethics approval. Second, the IRBs review the protocol and send the protocol with an accompanying approval letter to the federal ethics review committee. Third, the received protocol is further reviewed by the NEC, and then sent to the RA for final approval and authorization. Taking this approval process into account, we asked study participants about their experiences in clinical trial review and approval processes to understand the feasibility of arranging PTA.

#### PTA responsibilities in clinical trial approval, monitoring, and follow-up

Some of the principal investigators and IRB members said that PTA arrangements were the responsibility of the sponsor, the principal investigator, ethics review boards and regulatory authorities. A principal investigator stated:

The sponsor and the investigator should consider PTA from the beginning in the protocol and the informed consent, and the ethics committee and the regulatory authority should check these during the protocol approval process. (R7-PI3 participants)

Some principal investigators explained their role after completion of clinical trials as submitting the final reports, engaging in dissemination activities, and advocating for accessibility of the medicinal products. They said:

The researcher’s main role and responsibility after trial completion is to submit the final report, develop a policy brief, and arrange a dissemination workshop (R12-PI4 participant).

Suppose it is a new drug efficacy test, and the outcome is positive. In this case, the principal investigator can deal with the company to start its business in the country to ensure accessibility with a reasonable price and facilitate its feasibility in urban and rural areas. (R13-PI5 participant)

In explaining their PTA responsibilities, participants from the RA stated that the role of their office was to focus on arrangements of health insurance for the study participants, risks mitigation options, and dissemination of the study results. The dissemination of study findings (knowledge) provided input for different programs and service improvements, such as changes in treatment protocols or modifications to procedures, as explained in the excerpt below.

If the trial is on neglected tropical diseases (NTD), the result should be communicated to people from the NTD program if they need to change their treatment protocol or make modifications. We give them a recommendation on what to change, and based on that, there will be changes in policy or procedure. For instance, the HIV drug was given from different drugs regimens of varying doses, but that was changed to a single dose treatment based on the research results. (R4-RA participant)

For clinical trials follow-up, participants from the NEC and an IRB said that they have a limited role in monitoring and following up trials approved by their offices. They stated that the RA is responsible for trial monitoring and follow-up as they are the ultimate decision makers in the approval and authorization process.

One of the IRB participants elaborated:

The regulatory authority evaluates the product, registers it and oversees the process of the conduct of the trial, and they are the legal entity vested with authoritative power to monitor and even cancel the trials if not conducted according to the protocol. We work more from the moral obligation and professional perspectives of looking at ethics; we do not have authoritative power to stop the trials if we find mistakes. (R8*-*IRB3 participant)

We inquired about the clinical trials follow-up process from RAs; they stated that they relied on investigators’ mid-term and annual reports and rarely visited study sites. A RA participant said:

We mainly rely on the investigators’ mid-term and annual reports for monitoring and follow-up of trials, we rarely visit trial sites. (R4*-*RA participant)

### Feasibility of PTA arrangements and challenges

Most of the study participants were uncertain about the feasibility of arranging PTA because of communication gaps and poor collaboration between principal investigators and public health institutions, lack of guidelines, regulations, policies, and health acts, poor community engagement in clinical trial approval processes and fear of lack of donor support when PTA requirements are put in place.

Some participants from the NEC, RERC and IRBs indicated that there were significant communication gaps and poor collaboration amongst principal investigators, public health institutions and the community regarding trials and their benefits to the community. An RERC member elaborated:

There is a significant communication gap and collaboration between the principal investigators and public health institutions on the shared understanding about the trials conducted in their area and related benefit. (R15-RERC2 participant)

The limited number of clinical trials, lack of transparency about the clinical trials, and lack of harmonized working processes between the regulatory authority and national research ethics review committee, delayed protocol approval, and donor-driven research with little relevance to the country’s health problems could limit the practicality of PTA implementation. (R9-NEC participant)

Poor stakeholder collaboration specifically community engagement in study design, implementation and dissemination of study knowledge and products hindered meaningful PTA. Participants stated:

Community engagement is very less or partial, and even if they engaged during the study period, their access to the trial information is low after the study is completed. For instance, results might be presented as a policy brief, recommendations, and reports for concerned stakeholders and organizations, and published in peer-reviewed journals, but communities may not have access to the expected benefits. (R12-PI4 participant)

PTA arrangements must involve stakeholders before the study begins. (R3-FO participant)

Consequently, it was proposed that a discussion forum for the sponsors, principal investigators, research ethics committees and regulators, and community be established for a shared understanding of health problems, ethical issues, and PTA arrangements to be created.

Most study participants stated that lack of guidelines, regulations, policies, and health acts enforcing PTA were a potential hindrance to PTA implementation. Participants said:

In the absence of a guiding document, requesting sponsors for the PTA arrangements cannot be realistic. We understand study participants should be the primary beneficiaries of a successful trial, but we do not have an article concerning PTA arrangements and implementations in the current working guidelines. (R17-PI6 participant)

For example, we have a law for material transfer agreement (MTA) for genetic material, but it does not include human; so, the declaration of Helsinki must be interpreted considering local contexts. (R16-IRB5 participant)

To address the challenges related to laws and regulations, participants from RAs said that they needed to improve the working documents by creating and implementing directives or regulations which can help to enforce PTA. An IRB member recommended the establishment of a PTA coordination office to facilitate implementation. The participant said:

In my opinion, the regulatory authority (the FDA) could be the right office to establish a PTA coordination office from different disciplines, in consultation with the ethics review committee and other relevant professional for an effective PTA implementation. (R8-IRB3 participant)

It was recommended that a PTA guideline is developed to guide implementation. A principal investigator elaborated:

PTA arrangement guidelines must be developed to guide implementers, and serve as a legal document, to follow, support, and enforce the implementing agency when needed. (R7-PI3 participant)

Participants were hesitant to discuss PTA with donors and researchers due to the resultant additional costs. They opined that donors and researchers might be unhappy with the arrangements and cancel planned clinical trials. A principal investigator said:

Principal investigators and donors may not feel happy if they are requested to include PTA with an additional budget. (R13-PI5 participant)

## Discussion

The study findings highlight the perspectives of diverse stakeholders essential to PTA arrangements and implementation. The results demonstrate significant variations in how PTA is understood, particularly regarding describing PTA, beneficiary identification, procedural requirements, and implementation challenges. Perspectives emerged from study participants operating in low-resource settings, where weak health systems, infrastructural limitations, and inadequate research policies and regulations indicate the feasibility challenges for effective PTA implementation. These insights underscore the need for context-specific strategies to address stakeholder discrepancies and the systemic constraints required for PTA implementation frameworks.

The study findings revealed that participants had limited or inconsistent understandings of PTA. These findings are similar to the study on PTA in maternal vaccines which found that principal investigators were not fully aware of the concept of PTA (Van Roessel et al., 2019). The few who understood it in the study linked it to continuity and availability of drugs to research participants after completion of clinical trials. This is of course a welcome finding. However, as we saw in the introduction, PTA is not limited to continuity and availability of drugs. PTA has other dimensions including the availability and accessibility of knowledge to the population and host country; availability and accessibility of the study drug and of associated medical care an infrastructure to research participants who might still need the intervention after the trial; arrangements for availability and accessibility of the intervention in the community and/or host country. If PTA is to be successfully implemented in Ethiopia, there is a real need for training of principal investigators, sponsors, research ethics committee members and RA members to better understand the clinical trial design involving PTA planning and implementation.

Regarding PTA beneficiaries, there were conflicting responses from the research participants as some thought only the clinical trial participants should benefit from successful interventions whereas others thought the whole community should benefit. Some researchers advocate for research participants within clinical trials to benefit from PTA (Cho et al., 2018; Doval et al., 2015). The CIOMS guidelines, as well Kurihara, Greco and Dhai, among many others, argue that PTA to proven interventions should not only be ensured for trial participants but available and affordable for those in need nationally and globally (CIOMS, 2016; Kurihara et al., 2023). These arguments may undermine the need for real beneficiaries and increase their exploitation. It is crucial for trial participants to have continued access to proven products or procedures after the study concludes. Additionally, making these interventions available to host countries and to other LMICs in need of intervention will help reduce disparities in access for marginalized and underserved communities.

Several research participants believed that ensuring PTA was a shared responsibility among principal investigators, sponsors, research ethics committees and regulatory authorities. While the role of principal investigators and sponsors were clearly described such as disseminating study findings and facilitating access to investigational products, the specific responsibility of ethics review committee members remained unclear.

The research findings indicate that the clinical trial review and approval process in Ethiopia involves multiple tiers, including IRB, national ethics committee and finally the RA. This multi-layered system may create ambiguity regarding responsibility for ensuring PTA to interventions. Notably, RA members acknowledged their role in knowledge dissemination but cited limited capacity for monitoring and following up approved clinical trials due to financial constraints. In a study on PTA views among study participants and stakeholders in South Africa, research participants listed sponsors, principal investigators, research participants, and the government as parties responsible for PTA (Ngwenya et al., 2022). According to the study, the government’s responsibility is to ensure treatment is available to all patients at the end of the trial.

Opinion’s on sponsor’s responsibilities for PTA were divided among participants: some argued that sponsors should not bear this obligation while others contended that they should be responsible. In contrast, principal investigators were consistently expected to uphold harm prevention measures and ensure that the communities derived tangible benefits from research. Regarding responsibilities for PTA, the CIOMS guidelines explicitly state that ensuring PTA constitutes an ethical obligation for both researchers and sponsors to be fulfilled in collaboration with government authorities and other relevant stakeholders (CIOMS, 2016). The divergent understandings among relevant stakeholders regarding PTA obligations must be addressed through ongoing training programs and the development of clear implementation guidelines. Without such measures, the practical feasibility of ensuring meaningful PTA may remain uncertain.

The research findings revealed that research participants expressed uncertainty about the feasibility of PTA. This uncertainty stems from the fact that Ethiopia lacks the necessary guidelines, regulations, policies, and health acts to support the PTA process. As a results, regulators do not have a legal foundation for enforcing PTA. Our literature review indicates that most Sub-Saharan African countries with the exception of South Africa also lack legislation and specific guidelines on PTA. To facilitate PTA, research participants recommended establishment of PTA legislation and guidelines in Ethiopia.

In contrast, research ethics committee and RA members expressed concern that PTA obligations may deter sponsor participation in clinical trials due to increased operational costs. This is a shared concern among some researchers who argue that obligating pharmaceutical companies and sponsors to ensure PTA will deter sponsors or limit the number of clinical trials due to increased costs and affect overall profits (Doval et al., 2015; Mehrotra & Manchikanti, 2024; Singh et al., 2019; WMA, 2013). To reduce high costs incurred by sponsors and pharmaceutical companies, it has been suggested that governments should provide incentives such as tax or fee exemptions and fast-track approval of beneficial drugs/interventions (Singh et al., 2019). Additional recommendations include pharmaceutical companies supporting PTA through corporate social responsibility initiates, allocating surplus clinical trial funds, and establishing an international fund with philanthropic support (Naidoo et al., 2021). These alternative cost-reduction mechanisms could enhance the feasibility of implementing PTA, particularly in resource-constrained settings across sub-Saharan Africa where clinical research predominantly relies on foreign funding.

Study participants raised concerns about poor stakeholder engagement, particularly in community engagement, during the design, implementation and dissemination of clinical trial findings. This lack of engagement could limit meaningful planning and implementation of PTA. According to CIOMS guidelines, communities should be actively engaged in the planning and arrangements for PTA (CIOMS, 2016). Research participants recommended a establishing a multi-stakeholder forum involving sponsors, principal investigators, research ethics committees and regulators, and community representatives. Such a platform would foster a shared understanding of health priorities, ethical considerations, and PTA arrangements. Furthermore, participants emphasized comprehensive PTA planning with active stakeholder engagement is essential for: (1) facilitating equitable negotiations, (2) ensuring tangible community benefit and, (3) prevent potential exploitation of research participants and the host community in clinical trials. These findings align with existing literature emphasizing the importance of collaborative research approaches to ensure meaningful PTA (Grady, 2005; Kurihara et al., 2023; Van Roessel et al., 2019). These studies have demonstrated that multi-stakeholder engagement and partnership significantly enhance the feasibility and sustainability of PTA arrangements.

This study substantially advances understanding of PTA in low-resource settings of Sub-Saharan Africa, where fragile healthcare systems intersect with underdeveloped research governance system. The findings underscore the occurrence persistent inequities in research benefit-sharing, particularly the systematic neglect of PTA as an ethical obligation. This oversight has resulted in widespread deprivation of meaningful benefits for research participants and host communities, despite their indispensable contributions to scientific progress. Moreover, these findings call for urgent scholarly attention to amplify the need for ethical obligations to include PTA in global health research.

### Strengths and limitations of the study

This study strength lies in exploring an under-researched area in sub-Saharan Africa, specifically Ethiopia. We interviewed several key stakeholders involved in clinical trials including research ethics committee members, RA members, principal investigators and a funder providing an enriched discussion on PTA knowledge and feasibility in Ethiopia. Purposive sampling and snowballing sampling select participants based on predefined criteria, offering targeted insights although risking researcher bias and limited generalizability. Both approaches are valuable for qualitative research but lack statistical representativeness, making them ideal for exploratory expert-driven in-depth investigations. However, our findings are transferable to similar contexts. In addition, the study could have benefitted from the voices of trial participants and community members.

## Data Availability

The interview transcripts contain personal data and do not have approval from the ethics committee for open data sharing.
